# Aging in Bangladesh: A Wake‐Up Call for Elderly Care Solutions

**DOI:** 10.1002/hsr2.70529

**Published:** 2025-02-23

**Authors:** Shimpi Akter, Md Ikbal Hossain, Humayun Kabir, Sopon Akter, Masuda Akter

**Affiliations:** ^1^ Department of Medical Studies Bangladesh University of Professionals Dhaka Bangladesh; ^2^ Faculty of Nursing University of Alberta Edmonton Alberta Canada; ^3^ Department of Public Health North South University Dhaka Bangladesh; ^4^ Begum Rabeya Khatun Chowdhury Nursing College Sylhet Bangladesh; ^5^ School of Medical Sciences Shahjalal University of Science and Technology Sylhet Bangladesh; ^6^ Department of Health Research Methods Evidence and Impact, McMaster University Hamilton Ontario Canada; ^7^ Department of Economics American International University‐Bangladesh Dhaka Bangladesh; ^8^ Faculty of Medicine University of Dhaka Dhaka Bangladesh

**Keywords:** aging, Bangladesh, elderly, home care, residential care, senior housing

## Abstract

**Background and Aims:**

Bangladesh has been experiencing a rapid demographic shift from the younger population to older adults, with the older adults facing significant challenges due to the absence of residential and formal home care models. This perspective aimed to evaluate the status of residential and formal home care models and propose potential strategies to improve elderly care in Bangladesh.

**Methods:**

This perspective utilized a narrative review approach to search existing literature on elderly care in Bangladesh on PubMed, Google Scholar, and Web of Science. The perspective reviewed and presented the status of elderly care and identified gaps in residential and formal home care infrastructure by analyzing existing literature.

**Results:**

In Bangladesh, there was no evidence found that any authority had ever initiated or evaluated residential and formal home care programs, showing systematic neglect that resulted in the suffering of older adults. The collapse of the traditional informal family care model, fueled by urban migration and shifting socioeconomic dynamics, has further isolated older adults, leaving them with considerable unmet care needs. The issue is being worsened by a high prevalence of chronic illnesses such as diabetes, hypertension, and cardiovascular disease, as well as the collective inability to meet the growing demand for elderly care. Furthermore, limited access to healthcare services, particularly in rural areas, emphasized the urgent need for comprehensive elderly care models in Bangladesh.

**Conclusion:**

The proposed strategies involve developing residential care facilities, probably on a small scale, and integrating formal home care services in Bangladesh. However, conducting preliminary investigations is essential, including investigating the model to be implemented, training caregivers, and establishing financial support programs and regulatory frameworks. Thus, the government and private sector organizations should collaborate to address these challenges of older adults in Bangladesh.

## 1 Background

Aging is an inevitable biological process affecting all individuals; however, the aging experience can vary significantly depending on the policies and residential care infrastructure to support the older adults [1]. In Bangladesh, a developing country where residential and formal home care models to support older adults have historically been absent, the aging experience is particularly arduous [2]. With a population exceeding 160 million in a geographically small country, the demographic profile remains predominantly youthful, with half of the population under 18 years of age [3]. Nonetheless, this youthful demographic is projected to age rapidly, placing Bangladesh on the cusp of a substantial demographic transition. Currently, a considerable portion of the population is classified as older adults, and this proportion is expected to grow significantly in the near future [4]. Unfortunately, there is an absence of a formal care model, such as nursing homes, including long‐term care (LTC) for individuals with significant dependency, and senior housing options—including retirement homes, assisted living facilities, and independent living arrangements—designed to support those with moderate dependency. This absence of any kind of formal care model leaves Bangladesh unprepared to address the impending demographic challenges of its future older population. In this perspective, we reviewed the current status of early care and potential strategies for elderly care, focusing on building sustainable residential and formal home care models to ensure the safety, well‐being, and dignity of older adults in Bangladesh.

## 2 Methods

This perspective utilized a narrative review approach to search existing literature on elderly care in Bangladesh on PubMed, Google Scholar, and Web of Science. The perspective aimed to assess the current status of elderly care and identify gaps in residential and formal home care infrastructure by analyzing evidence from peer‐reviewed articles and reports. This perspective also aimed to advocate for actionable strategies for developing sustainable residential and formal home care models for older adults in Bangladesh.

## 3 Aging Is a Growing Concern in Bangladesh

In Bangladesh, aging is an increasingly significant concern due to the potential demographic shift from younger to older adults, and this rise is burdened with chronic diseases such as diabetes, hypertension, and cardiovascular conditions [4]. The burden of this shift is possible to mitigate and improve the overall life expectancy of the older adults while the challenges of elderly care are being managed effectively. However, as of today, Bangladesh has no regulated residential care model that includes a range of care settings such as retirement homes, assisted living facilities, LTC, nursing homes, and so on [5]. Moreover, there is also no formal home care model available, leaving the entire older population without any form of formal care support as they age.

## 4 Profile of the Older Adults in Bangladesh

It was estimated that by 2050, the older adults aged 60 years and more will rise from 6% in 2001 to over 20%, with the absolute number increasing from 8 million to 44 million in Bangladesh [6]. Barikdar et al. reported that the older adults aged 60 years and more in Bangladesh increased from 1.38 million in 1974 to 7.59 million in 2011 [5]. The challenges vary significantly between the rural and urban settings as the health access for the older adults in Bangladesh is highly skewed toward urban settings [7]. The older adults in rural area face unique challenges influenced by cultural, economic, and social forces [8]. Cain reported that approximately 91% of older adults live with or next to a mature son, but this proportion decreases among the economically poor [8]. The majority of older adults (55.7%) in rural Bangladesh were reported to be on average health, with 20.3% in poor health, and their health was shown to be substantially connected with age, education, income, sanitation access, family structure, and living arrangements [9]. Islam et al. reported that approximately 67% of the aged in Pabna district reported suffering from at least one disease in the previous 3 months, with common health issues including high blood pressure, joint discomfort, body aches, heart problems, and vision problems [10]. The study also reported a strong association between health issues and socioeconomic position, underlining the difficulties influenced by limited healthcare financing and the lack of a comprehensive social assistance system, particularly in rural areas [10]. According to a recent study, the health problems among the older adults observed included general weakness and numbness of limbs (31.57%), musculoskeletal issues (16.44%), eye problems (11.84%), gastrointestinal issues (9.86%), respiratory problems (7.89%), cardiovascular issues (8.55%), ENT problems (9.86%), and urogenital issues (9.21%) [11].

## 5 Current Status of Elderly Care Model in Bangladesh

From the perspective of developed countries such as the United States or Canada, which have various residential or formal home care models, it may be difficult to comprehend the harsh reality faced by the older adults in Bangladesh [12]. The main concern may be the failure of Bangladesh to acknowledge the significant demographic shift it is experiencing and its lack of care models to meet these challenges. Unfortunately, no literature indicates any initiatives, activities, or involvement in investigating were carried out by any authorities on these aging issues and attempting to provide a solution. Numerous issues, such as cost‐effectiveness versus GDP of the country and priorities, may be behind these non‐invitations. While the residential care model may be deemed too costly, could Bangladesh adopt a model better suited to the context? This question raises worry as no initiative has been observed until today. As a result, older adults appear to be systematically neglected in Bangladesh. This neglect is reprehensible and necessitates immediate action to ensure a sufficient care model and support system for the older adults.

## 6 What Are the Residential and Formal Home Care Models?

Residential care includes the care settings where seniors transition from their own homes to residential environments that address their unmet care needs. Individuals with significant cognitive impairments, functional limitations, or major chronic diseases typically transition to high‐dependency settings such as nursing homes or LTC facilities [13]. Conversely, those with mild cognitive impairments, functional limitations, or social needs often transition to moderate dependency settings, such as retirement homes, assisted living facilities, senior housing, or independent living arrangements [13]. Bridging the gap in care, home care services provide support, sometime mild to comprehensive care, in residents' own homes as well as in residential care settings. The home care model may be often effective in potentially delaying or preventing the need for a transition to residential care settings.

## 7 Is the Traditional Family Support Model Changing?

Although certain forms of family care, known as informal home care for older adults provided by family members in the extended family [14], may persist, they have been considerably impacted by the erosion of the conventional family support model in recent decades, maybe influenced by urban migration and evolving family dynamics in Bangladesh [5]. This change has frequently left older adults, mostly in rural areas, alone or isolated in their own homes [15]. These changes in the family support model and a lack of residential and formal home care models have compounded the issues in Bangladesh. Therefore, today, older adults experience fear of being isolated and uncertain, regardless of their economic status. They suffer in silent despair, clearly ignored by the system that fails to provide any form of support in their final years of life.

## 8 How Do the Changes in Traditional Family Support Impact Elderly Care?

Traditionally, the responsibility of caring for elderly parents in Bangladesh has fallen to sons and daughters‐in‐law [16]. Daughters are generally unable to provide informal care for their elderly parents because they typically leave their parents' homes after marriage and transition to their husbands' homes. However, this traditional family support model has been in change as more young adults migrate to urban areas in pursuit of education, employment, and a better lifestyle [17]. This societal shift has left many older parents in rural areas without adequate informal care or family support, particularly as sons and their families no longer reside with them. According to Luna, older adults in Bangladesh often perceive a lack of sufficient care, with factors such as poverty, widowhood, and the migration of sons contributing significantly to the care deficit [2]. Rahman et al. emphasize that the presence of sons and their wives substantially reduces mortality among elderly men and women in Bangladesh [16]. As urban migration increases and breaks the traditional family support model, elderly parents face significant isolation and emphasize the urgent need to think about adopting any form of residential and formal home care solutions.

## 9 When Should We Take the Initiative to Address Infrastructural Gaps?

According to Kabir et al. (2016), with the growing older adults and the breakdown of the traditional family care model in Bangladesh, the absence of any formal residential and home care model has substantially impacted older adults, leaving them with serious concerns [18]. Our review of the literature reveals no evidence that any initiatives were taken to develop any formal care model for the older adults in Bangladesh. It should be counted as unfortunate that little to no progress has been made in this issue of any formal care model to address the unmet care demands of the older adults since the born of Bangladesh in 1971 [19]. As 2024 come to an end, the question of how long older adults in Bangladesh will have to wait for significant action emerges. The time has already come for the public and private sectors to work together to investigate and put into practice ideas for providing the older adults with necessary care. It is no longer fair to disregard the needs of the older adults, especially because Bangladesh has committed to achieving a number of Sustainable Development Goals (SDGs) put forward by the UN. These challenges offer an opportunity to adopt an effective care model from other nations or develop a care model specific to the context of Bangladesh. It is essential to increase awareness of the value of specialized and regulated residential and formal home care models. To ensure a comprehensive and sustainable strategy, the government should take the lead in developing these care models, maybe with active involvement and support from industry and non‐profit organizations.

## 10 Actions to Develop Residential and Formal Home Care Models

The suffering of the seniors in Bangladesh is prolonged when elderly care is neglected, denying them the dignity and support they deserve in their final years [5]. The study reported that there is significant residential and formal home care demands in Bangladesh [15]. Still, a planned strategy would be beneficial to reduce the burden of huge residential care needs, beginning with establishing small‐scale residential care facilities for individuals in dire need and investigating the feasibility of home care services as an alternative. Home care support services alongside residential care may provide comprehensive medical, personal, and social assistance, significantly improving the quality of life. Even if established programs are absent, it is important to establish a foundation in Bangladesh that includes generating baseline evidence, legislation, and regulations. To start with, specific safety standards and regulatory frameworks must be implemented to assure the safety and well‐being of residents while models are implemented. Researchers should focus on investigating this issue within the unique socioeconomic and cultural context of Bangladesh. These investigations can help to establish formal care solutions that are economically, culturally, and socially feasible. Simultaneously, public education campaigns are critical for raising knowledge about elderly care, influencing shifts in societal attitudes, and establishing support from society for the implementation of much‐needed care models. These collaborative activities may potentially establish the foundations for sustainable residential and formal home care systems in Bangladesh.

## 11 What Are the Potential Funding Sources for the Actions in Place?

Determining funding sources is one of the most difficult components of establishing residential and formal home care models. Some options would be for the government and nongovernmental organizations (NGOs) to collaborate to develop sustainable funding mechanisms. Even though Bangladesh is a developing country, it provides public healthcare coverage for all citizens and has regulated private healthcare coverage from the out‐of‐pocket cost [20]. Therefore, from the feasibility perspective, it may be possible to implement where a comparable plan for a formal residential care model is required. Special programs that provide financial assistance or subsidies for residential and formal home care services are crucial in making these options available to low‐income seniors yet may be limited to high‐income seniors. As it would be entirely new to Bangladeshi society if ever initiated to implement, the authorities should explore practical options for implementing such programs. In addition to funding, a qualified workforce is essential for providing high‐quality residential and formal home care services. Also, developing a competent workforce for residential and formal home care requires establishing educational and training programs for caregivers. Advantages include specialized training, which will not only ensure high‐quality care for the older adults but will also open up possibilities for employment in the elder care industry. In the end, it would not be an easy task, as establishing a residential and formal home care model in Bangladesh requires a combination of initiatives, including securing finances, providing financial help, and training the workforce. This approach would certainly address the growing demand for elderly care in Bangladesh, allowing the older adults to live with dignity, security, and the assistance they deserve.

## 12 Conclusion

Bangladesh is at a critical juncture in addressing the challenges posed by the rapidly growing older adults. The collapse of the traditional family care model, combined with the absence of regulated residential and formal home care models, has left the older adults isolated, vulnerable, and deprived of the support they deserve. Studies reveal that a significant proportion of the older adult faces health issues, with conditions such as musculoskeletal problems, cardiovascular diseases, and vision impairments being common, especially in rural areas where access to healthcare remains limited. To address these challenges, a comprehensive and long‐term structure that includes residential care facilities as well as integrated home care services is required. Some preceding work may be required, such as caregiver training, regulatory standards, and flexible funding sources. This is more than just fulfilling a moral obligation; it is a strategic investment in the social fabric of the country and demonstrates commitment of Bangladesh to global goals, including the United Nations SDGs. Bangladesh can adopt or develop care models appropriate for its socioeconomic and cultural establishment by encouraging collaboration among the government, the private sector, non‐profit organizations, and NGOs. The time to act is now such as small‐scale experimentation programs, raising awareness about elderly care, and training professionals can all serve as starting points for developing an effective care model. As these efforts grow, they will not only improve the lives of the older adults but also establish intergenerational solidarity, ensuring that aging is viewed as a period of life to be celebrated and supported. Ultimately, investing in elderly care will reflect principles of dignity a society and respect for its older population. With vision, commitment, and collaborative efforts, Bangladesh can transform its approach to aging, ensuring that its older population can live their final years with dignity, security, and purpose, ushering in a more promising future for generations to come.

## 13 Recommendation

There is currently a significant gap in baseline research on the demographics of older adults in Bangladesh, which drives an urgent need for research investment to better understand their profiles and current status of care. Future research should explore the shifts in informal family care models, assess the needs and expectations of older adults, and evaluate the feasibility of establishing residential and formal home care models in the country. This may include adapting care models that align with the unique social and cultural context of Bangladesh. Approaches such as incorporating the guidance of World Health Organization on LTC for universal health coverage, research should focus on developing interventions that address local needs and resources [21]. These efforts may be able to bridge the substantial gaps in residential and formal home care models to foster a sustainable and compassionate approach to elderly care in Bangladesh.

Furthermore, the government must take the lead, with researchers, policymakers, and funders actively collaborating to recognize and address these critical issues. Stakeholders need to acknowledge the urgency and significance of the problem. Raising awareness could be achieved by incorporating elderly care and its importance into school curricula and encouraging universities to invest in research and academic programs focused on elderly care. Practical interventions should include building retirement homes, establishing LTC facilities, and providing formal home care services for those in need. Therefore, prioritizing these measures may be essential in creating a supportive and sustainable framework for elderly care.

Certainly, funding remains a critical aspect of the discussion. While investments are made in other sectors, prioritizing aging can build unique investment opportunities. The role of the private sector in contributing to funding and the extent of its involvement must be carefully considered. This raises the importance of establishing clear regulations to govern these initiatives—defining how systems will be managed and outlining the laws and policies that will guide them. At this stage of its development, introducing residential and formal home care models may represent ambitious step for many, but it could be an important step to uphold the dignity and well‐being of its aging population in Bangladesh.

## Author Contributions

**Shimpi Akter:** conceptualization, investigation, writing – original draft, writing – review and editing, methodology, resources, project administration, validation, visualization. **Md Ikbal Hossain:** methodology, conceptualization, investigation, writing – original draft, writing – review and editing, visualization, project administration, resources. **Humayun Kabir:** conceptualization, investigation, writing – original draft, writing – review and editing, methodology, supervision. **Sopon Akter:** conceptualization, investigation, writing – original draft, writing – review and editing, resources, visualization, validation. **Masuda Akter:** writing – original draft, writing – review and editing, visualization, validation, methodology, conceptualization, investigation, project administration, supervision.

## Conflicts of Interest

The authors declare no conflicts of interest.

## Transparency Statement

The lead author Shimpi Akter affirms that this manuscript is an honest, accurate, and transparent account of the study being reported; that no important aspects of the study have been omitted; and that any discrepancies from the study as planned (and, if relevant, registered) have been explained.

## Data Availability

Data sharing not applicable to this article as no datasets were generated or analyzed during the current study. The authors confirm that no original data were collected for this work. All relevant literature used for this work is referenced within the article. Shimpi Akter has full access to all of the articles included in this work.

## References

[1] N. Szczygiel and M. Almeida , “Housing Policies for the Elderly: Why Should We Care?,” Public Policy Adm 16 (2017): 583–592, 10.13165/VPA-17-16-4-05.

[2] F. F. Luna , “Perceived Availability of Care and Support Among Older Adults in Bangladesh,” Innovation in Aging 3 (2019): S676–S677, 10.1093/GERONI/IGZ038.2500.

[3] S. Matubbar and Md Arifuzzaman , “Human Rights Violation of the Child and the Legal Protection in Bangladesh,” Asian Journal of Social Sciences and Legal Studies 4, no. 6 (2022): 223–232, 10.34104/AJSSLS.022.02230232.

[4] A. R. Sarker , I. Zabeen , M. Khanam , R. Akter , and N. Ali , “Healthcare‐Seeking Experiences of Older Citizens in Bangladesh: A Qualitative Study,” PLOS Global Public Health 3 (2023): e0001185, 10.1371/JOURNAL.PGPH.0001185. 36962985 PMC10022267

[5] A. Barikdar , T. Ahmed , and S. P. Lasker , “The Situation of the Elderly in Bangladesh,” Bangladesh Journal of Bioethics 7 (2016): 27–36, 10.3329/BIOETHICS.V7I1.29303.

[6] R. Kabir , H. T. A. Khan , M. Kabir , and Md. T. Rahman , “Population Ageing in Bangladesh and Its Implication on Health Care,” European Scientific Journal 9 (2013): 34–47.

[7] J. M. Abdullah , M. M. Ahmad , and S. E. Saqib , “Understanding Accessibility to Healthcare for Elderly People in Bangladesh,” Development in Practice 28 (2018): 552–561, 10.1080/09614524.2018.1450845.

[8] M. T. Cain , “The Activities of the Elderly in Rural Bangladesh,” Population Studies 45 (1991): 189–202, 10.1080/0032472031000145386.

[9] T. Uddin , M. A. I. Chowdhury , M. N. Islam , and G. U. Baher , “Status of Elderly People of Bangladesh: Health Perspective,” Proceedings of the Pakistan Academy of Sciences 47 (2010): 181–189, https://paspk.org/wp-content/uploads/proceedings/a22e165bproc47-3-7.pdf.

[10] S. Islam , R. Islam , F. Hossain , and P. K. Karmokar , “Socio‐Economic Status and Health Issues of Older Adults in District Pabna, Bangladesh,” Journal of Social Sciences 18 (2022): 84–94, 10.3844/JSSP.2022.84.94.

[11] H. Kabir S M H , “Health Problems of the Elderly Attending Tertiary Level Hospital in Urban Area,” Journal of Diabetic Association Medical College, Faridpur 4 (2020): 26–30, 10.70357/JDAMC.2020.V0401.07.

[12] United Nations Fund for Population Activities , European Demographic Information Bulletin 3 (1972): 107–108, 10.1007/BF02917926.

[13] H. Kabir and A. Costa , *Economic Status and Transitions to Seniors' Housing: An Analysis Using the Canadian Longitudinal Study on Aging* (2024), https://macsphere.mcmaster.ca/handle/11375/30608.

[14] D. Boucaud‐Maitre , M. Cesari , and M. Tabue‐Teguo , “Foster Families to Support Older People With Dependency: A Neglected Strategy,” Lancet Healthy Longevity 4 (2023): e10, 10.1016/S2666-7568(22)00288-4. 36610444

[15] H. Khan and G. Leeson , “The Demography of Aging in Bangladesh: A Scenario Analysis of the Consequences,” Hallym International Journal of Aging 8 (2006): 1–21, 10.2190/HA.8.1.A.

[16] O. Rahman , J. Menken , and R. Kuhn , *The Impact of Family Members on the Self‐Reported Health of Older Men and Women in a Rural Area of Bangladesh* (2004), 10.1017/S0144686X04002314.

[17] M. I. Rahman and A. M. Ali , *Population Aging and Its Implications in Bangladesh* (2009), https://papers.ssrn.com/abstract=1349447.

[18] R. Kabir , M. Kabir , M. S. Gias Uddin , N. Ferdous , and M. R. Khan Chowdhury , “Elderly Population Growth in Bangladesh: Preparedness in Public and Private Sectors,” IOSR Journal of Humanities and Social Science 21 (2016): 58–73, 10.9790/0837-2108025873.

[19] M. N. Islam and D. C. Nath , “A Future Journey to the Elderly Support in Bangladesh,” Journal of Anthropology 2012 (2012): 1–6, 10.1155/2012/752521.

[20] M. S. Pavel , S. Chakrabarty , and J. Gow , “Cost of Illness for Outpatients Attending Public and Private Hospitals in Bangladesh,” International Journal for Equity in Health 15 (2016): 167, 10.1186/S12939-016-0458-X. 27724955 PMC5057498

[21] World Health Organization , *Long‐Term Care for Older People: Package for Universal Health Coverage* (2023).

